# A Sensitive Method for Detecting *Beauveria bassiana*, an Insecticidal Biocontrol Agent, Population Dynamics, and Stability in Different Substrates

**DOI:** 10.1155/2023/9933783

**Published:** 2023-08-25

**Authors:** Zepei Gu, Lijie Chen, Weixing Zhang, Pin Su, Deyong Zhang, Xiaohua Du, Qianze Peng, Zhuoxin Liu, Xiaolan Liao, Yong Liu

**Affiliations:** ^1^College of Plant Protection, Hunan Agricultural University, Changsha 410128, China; ^2^Hunan Plant Protection Institute, Hunan Academy of Agricultural Sciences, Changsha 410125, China; ^3^Longping Branch, Graduate School of Hunan University, Changsha 410125, China

## Abstract

*Beauveria bassiana* is a well-known insecticidal biocontrol agent. Despite its broad field applications, its survival, colonization, and stability under field conditions remained unclear, mainly due to the lack of a quick and reliable detection method. In this study, we developed a quantitative real-time PCR technology to monitor the stability and population dynamics of *B. bassiana* in different substrates (water, soil, and on the cotton leaves surface), different spores of *B. bassiana* applied on Chinese cabbage leaves surface, and the lethality of *Pieris rapae* spraying with different spores of *B. bassiana*. Our results showed a decreased concentration of *B. bassiana* DNA in all three substrates from the 1^st^ day till 9^th^ day of post inoculation (dpi) period, possibly due to the death of *B. bassiana*. After this decrease, a quick and significant rebound of *B. bassiana* DNA concentration was observed, starting from the 11^th^ dpi in all three substrates. The *B. bassiana* DNA concentration reached the plateau at about 13^th^ dpi in water and 17^th^ dpi in the soil. On cotton leaves surface, the *B. bassiana* DNA concentration reached the highest level at the 17^th^ dpi followed by a small decline and then stabilized. This increase of DNA concentration suggested recovery of *B. bassiana* growth in all three substrates. We found that the most suitable killing effectiveness of *P. rapae* was the 1.0 × 10^7^ spores/mL of *B. bassiana.* In summary, we have established a detection technology that allows a fast and reliable monitoring for the concentration and stability of *B. bassiana* under different conditions. This technology can benefit and help us in the development of proper management strategies for the application of this biocontrol agent in the field.

## 1. Introduction

*Beauveria bassiana* is an entomopathogenic fungus that can infect more than 700 insect species belonging to 149 different families in 15 different orders by causing white muscardine disease in them [1–3]. To date, entomopathogenic fungi have been used as biological control agents for the management of various insect and mite pests [4]. *B. bassiana* is the most well studied entomopathogenic fungi and has been frequently used as a commercial mycoinsecticide in the field [5, 6]. For example, *B. bassiana* was used to control insect pests, including *Pissodes castaneus*, *Ostrinia nubilalis,* and *Rhynchophorus ferrugineus*, infield through direct spraying [7–9]. It is known that *B. bassiana* is safe to human, natural beneficial insects, and beneficial microorganisms. During field applications, it can also be used together with other commercial chemical pesticides [10–12]. More importantly, there is no evidence that insect pest(s) has evolved resistance against *B. bassiana.* Currently, *B. bassiana* is widely used to control insect pests in the order of Lepidoptera and Coleoptera, including many forest pests [13, 14].

It was reported that *B. bassiana* could secrete many biologically active compounds like lactide and poisonous proteases during its infection in insect hosts [15, 16]. *B. bassiana* is also known to hijack nutrients and water from the infected insects to accelerate their death process [17]. *B. bassiana* spores can be formulated to produce wettable powder for commercial use in agriculture as well as in forests [18–21]. A separate study conducted recently showed that the survival and successful recolonization of the *B. bassiana* in field depended largely on environmental factors [22]. We reasoned that the understanding of the survival and growth dynamics of this fungus in nature is necessary for the integration of this biocontrol agent into a more effective and safe plant protection strategy and to reduces public concerns on environment conservation [23, 24].

Peng and coworkers investigated the growth and survival of *Metarhizium anisopliae* in oriental migratory locust [25]. In this study, we used a similar strategy to establish an accurate quantitative real-time PCR technology to determine the stability of *B. bassiana* in various substrates (i.e., water, soil, and cotton leaves surface) at 28°C. With this new technology, we can now monitor *B. bassiana* population dynamics and regrowth in field samples and develop more effective management strategies using this and other biocontrol agents.

## 2. Materials and Methods

### 2.1. Materials

*B. bassiana* was originally isolated from a tomato field in Yueyang, China (113°00′4.39″E, 29°45′9.41″N) and then maintained in the laboratory. Before use, this fungus was grown till a concentration of 1.0 × 10^12^ spores/g·mass. The liquid growth medium used for *B. bassiana* was potato dextrose agar (PDA-Medium), and the carbon-to-nitrogen (C : N) ratio was at 10 : 1 (Vega. 2003). Cotton plants used in this study were grown in a greenhouse and the soil was from Chunhua in Changsha, China (113°25′56.50″E, 28°27′80.40″N), which contained 10% water, and the water used was the natural water from a lake in Changsha, China (113°09′04.77″E, 28°19′39.14″N).

### 2.2. DNA Extraction from *B. bassiana*

DNA was extracted from *B. bassiana* containing water, soil, and plant leaf samples using a DNA extraction kit (Tiangen Biochemical Technology, Beijing, China). Concentration and quality of each DNA sample were measured by using a spectrophotometer (Thermo Fisher Scientific, Massachusetts, USA). For each DNA sample, three technical replicates were analyzed during real-time fluorescent PCR with a SYBR Green I reagent (TransGen Biotech, Beijing, China) as instructed by the manufacturer (TransGen Biotech).

### 2.3. Synthesis of *B. bassiana* Specific Primers

The initial PCR amplification was carried out using a set of bacterial universal primers (Baker. 2003) and the DNA isolated from *B. bassiana*. The resulting PCR products were cloned and sequenced by the TSINGKN Biotech (TSINGKN Biotech, Beijing, China). Conserved DNA sequences obtained from the PCR products were analyzed using the DNAMAN software, and six pairs of real-time PCR primers were designed based on the obtained sequences using the Primer 5.0 software in the DNAMAN (LynnonBiosoft, USA). In the subsequent real-time PCR assays, diluted *B. bassiana* genomic DNA or double distilled water (ddH_2_O) were used as the positive and negative control sample, respectively. The six designed primer sets were first tested by gradient PCR with three different annealing temperatures (52, 54 and 56°C) and later by PCR amplifications. The resulting PCR products were visualized in agarose gels through electrophoresis. The primer set giving a single specific PCR product was considered to be the optimal *B. bassiana*real-time PCR primer set and was used in further experiments.

### 2.4. Preparation of Real-Time PCR Standard Curve

According to a previously described method [26], we first generated a recombinant plasmid DNA carrying a fragment of *B. bassiana* genomic DNA and then measured its concentration using a spectrophotometer. The copy number of the recombinant plasmid DNA was calculated as previously reported [27] and then further diluted prior to the real-time fluorescent PCR on a CFX96™Real-Time System (Bio-Rad, California, USA), and the standard curve was established using the concentration logarithm and the Ct values.

### 2.5. Determination of Detection Limit Using Purified *B. bassiana* DNA

*B. bassiana* was homogenized, dried, and diluted 1 : 99 (w/v) in sterilized water. The diluted sample was incubated at 28°C inside an incubator and sampled once every two days (one mL sample per sampling time) till the 29^th^ day of postincubation (dpi) period. The collected samples were centrifuged at 12,000 rpm/min for 1 minute and the supernatants were discarded prior to the extraction of DNA from the pellets. For real-time PCR reactions, three technical replicates were analyzed and used to represent a specific DNA sample.

### 2.6. Detection of *B. bassiana* DNA in Soil Samples

One gram dried *B. bassiana* powder was mixed with 99 grams sterilized black soil, incubated at 28°C in an incubator and then sampled once every two days (1 g soil sample per sampling time) as described above. The collected soil samples (0.1 g each) were used for DNA extraction as described above. Real-time PCR was then performed using three technical replicates per sample.

### 2.7. Detection of *B. bassiana* DNA in Cotton (*Gossypium* spp.) Leaves Samples

One gram dried *B. bassiana* powder was diluted in 99 grams sterilized water and then spread onto the surface of cotton leaves. After air drying, three to four cotton leaf pieces were randomly collected once every two days. The sampled cotton leaf tissues were grounded in liquid nitrogen and 0.1 g powder from each leaf sample was used for DNA extraction. For real-time PCR, three technical replicates were used to represent a specific sample.

### 2.8. Detection of Different Number Spores of *B. bassiana* DNA on Chinese Cabbage (*Brassica rapa Pekinensis*) Leaves

The *B. bassiana* with 1.0 × 10^2^ (A), 1.0 × 10^4^ (B), 1.0 × 10^6^ (C), 1.0 × 10^7^ (D), and 1.0 × 10^8^ (E) spores/mL were sprayed evenly on Chinese cabbage leaves at the same growth level at 28°C and 60% humidity. The buffer solution used for the dilution of the spores of *B. bassiana* was set as a blank control. The leaf samples were analyzed for *B. bassiana* DNA concentrations by real-time PCR after 24 hours of spraying, three to four pieces of cabbage leaf were randomly collected once every two days. For real-time PCR, three technical replicates were used to represent a specific sample.

### 2.9. Detection of Different Number Spores of *B. bassiana* DNA in *P. rapae*

The *B. bassiana* with 1.0 × 10^2^ (A), 1.0 × 10^4^ (B), 1.0 × 10^6^ (C), 1.0 × 10^7^ (D), and 1.0 × 10^8^ (E) spores/mL were used to spray uniformly on the cabbage leaves. After spraying 12 hours, cabbage leaves were fed to 40 heads of *P. rapae* selected at normal active fourth stage larvae, the *P. rapae* larvae were cultured in a net cage at 28°C and 90% humidity. The buffer solution used for the dilution of the spores of *B. bassiana* was set as a blank control. After feeding for 24 hours, one *P. rapae* were randomly collected to check for the *B. bassiana* DNA concentrations by real-time PCR. The selected *P. rapae* was rinsed with water to avoid the presence of *B. bassiana* outside the body, which will affect the experimental results. For real-time PCR, three technical replicates were used to represent a specific sample.

In this study, all the experiments were repeated twice to ensure the repeatability of the results.

## 3. Data Analysis

Data obtained from three independent experiments were combined and analyzed by using one-way ANOVA program in Excel (Microsoft® Office Excel 2003, USA) and the SPSS 13.0 statistical software package (SPSS, Inc., Chicago, IL, USA). Post hoc multiple comparisons were conducted at the 5% level of probability using Duncan's multiple range test (DMRT).

## 4. Results

### 4.1. Determination of Optimal PCR Primers and Standard Curve of Real-Time PCR

Gradient PCR was first used to determine the optimal annealing temperature for individual primer sets listed in supplementary Table 1. The recombinant plasmid DNA was diluted based on the copy number of plasmid DNA estimated using the following equation: copy numbers/*μ*L = (6.0 × 10^14^ copies × plasmid concentration (g)/*μ*L)/(number of bases × 660 Dalton/base). The efficiency of each primer set was determined through PCR reactions and the resulting PCR products were visualized in agarose gels through electrophoresis. Results of the assay showed that, when the annealing temperature was set at 54°C, four of the six primer sets gave strong and correct sized PCR product bands. At 52°C, three primer sets gave positive PCR products of different size, and at 56°C, only two primer sets gave positive PCR products (Supplementary Figure 1). Consequently, the 4^th^ primer set (e.g., 98F 5′GGCATCGATGAAGAACGCAG3′ and 333R 5′GTATTACTGCGCAGAGGTCG3′) was used for the following real-time PCR assays. Through PCR amplification assays, a standard curve of PCR was determined as *Y* = −3.26*X* + 11.25, *R*^2^ = 0.99965, and the PCR amplification efficiency = 1.03 (Supplementary Figure 2). Using serially diluted plasmid DNA samples, it was observed that by the 10-fold dilution of DNA samples; the Ct values increased by 3-4. The concentration of standard plasmid DNA was found to be closely correlated with the Ct values, leading to single melting curves (Supplementary Figure 3).

### 4.2. Stability of *B. bassiana* in Water at 28°C

**Figure 1:**
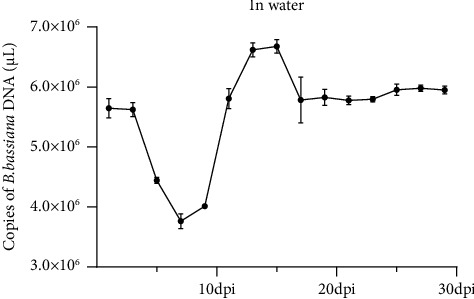
Stability of *B. bassiana* DNA in water and at 28°C. Three technical replicates were used for each sample and the experiment was repeated twice times. Statistical significances were determined by DMRT, *p* ≤ 0.05.

Dried *B. bassiana* powder was diluted in water and incubated at 28°C for several days followed by DNA isolation. Stability of *B. bassiana* DNA in water was determined as the concentrations of *B. bassiana* DNA in water over time by real-time PCR. The result of the assay showed that the amount of *B. bassiana* DNA in assayed samples stored for 1 and 3 days was similar (5.646 × 10^6^ and 5.622 × 10^6^ copies of *B. bassiana* DNA/*μ*L, respectively) (Figure 1). The amount of *B. bassiana* DNA decreased quickly to 3.762 × 10^6^ copies of *B. bassiana* DNA/*μ*L on the 7^th^ day of postinoculation period followed by an increase up to 6.619 × 10^6^ copies of *B. bassiana* DNA/*μ*L on the 13^th^ day of postinoculation period. *B. bassiana* DNA decreased again at the 17^th^ dpi and maintained at a similar level till 29^th^ dpi. In this study, no *B. bassiana* DNA was detected in the negative control sample.

### 4.3. Stability of *B. bassiana* DNA in Soil at 28°C

**Figure 2:**
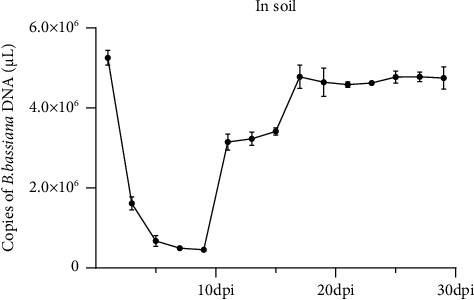
Stability of *B. bassiana* DNA in soil at 28°C. The stability of *B. bassiana* DNA was determined by qRT-PCR. Three technical replicates were used for each sample, and the experiment was repeated twice. Statistical significances were determined by DMRT, *p* ≤ 0.05.

Stability of *B. bassiana* DNA in soil was also determined by real-time PCR. Results shown in Figure 2 demonstrated that *B. bassiana* DNA concentration declined quickly from the 1^st^ dpi (5.253 × 10^6^ copies of *B. bassiana* DNA/*μ*L) to the 9^th^ dpi (4.530 × 10^5^ copies of *B. bassiana* DNA/*μ*L). As shown in Figure 2, *B. bassiana* DNA concentration rebound at the 11^th^ dpi and continued to increase till 17^th^ dpi (4.776 × 10^6^ copies of *B. bassiana* DNA/*μ*L). After this increase, *B. bassiana* DNA concentration remained relatively stable till 29^th^ dpi, suggesting a reestablishment of *B. bassiana* population in soil samples. This experiment was repeated twice.

### 4.4. Stability of *B. bassiana* DNA on Cotton Leaves at 28°C

**Figure 3:**
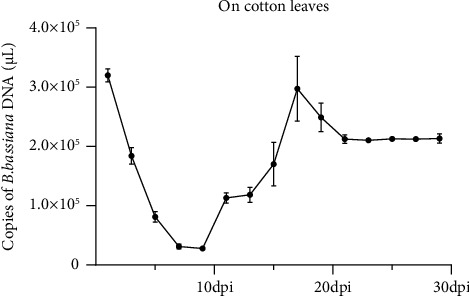
Stability of *B. bassiana* DNA on cotton leaves surface at 28°C as determined by qRT-PCR. Three technical replicates were used for each sample, and the experiment was repeated twice. Statistical significances were determined by DMRT, *p* ≤ 0.05.

Dried *B. bassiana* powder was diluted in water and sprayed onto the surface of cotton leaves. After incubation at 28°C for several days, the leaf samples were analyzed for *B. bassiana* DNA concentrations by real-time PCR. The results shown in Figure 3 indicated that the concentration of *B. bassiana* DNA was at 3.202 × 10^5^ copies of *B. bassiana* DNA/*μ*L at the 1^st^ dpi and then declined to 2.777 × 10^4^ copies of *B. bassiana* DNA/*μ*L at the 9^th^ dpi. The concentration of *B. bassiana* DNA rebound at the 11^th^ dpi and reached 2.975 × 10^5^ copies of *B. bassiana* DNA/*μ*L at the 17^th^ dpi. The *B. bassiana* DNA concentration decreased slightly again and reached 2.126 × 10^5^ copies of *B. bassiana* DNA/*μ*L at the 21^st^ dpi, and then remained stabilized. This experiment was repeated twice. *B. bassiana* DNA was not detected in the negative control samples.

### 4.5. Stability of Different Number Spores of *B. bassiana* DNA on Chinese Cabbage Leaves at 28°C

**Figure 4:**
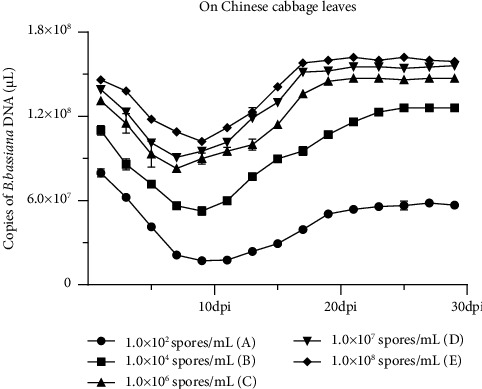
Stability of different number spores of *B. bassiana* DNA on cabbage leaves surface at 28°C as determined by qRT-PCR. Three technical replicates were used for each sample, and the experiment was repeated twice. Statistical significances were determined by DMRT, *p* ≤ 0.05.

To investigate the dynamics of different amounts of *B*. *bassiana* spores over time, we selected different concentrations of spores to spray on Chinese cabbage leaves and then detected the stability of *B. bassiana* DNA. Results shown in Figure 4 indicated that the concentration of *B. bassiana* DNA was at 7.976 × 10^7^ (A), 1.100 × 10^8^ (B), 1.308 × 10^8^ (C), 1.391 × 10^8^ (D), and 1.460 × 10^8^ (E) copies of *B. bassiana* DNA/*μ*L at the 1^st^ dpi and then declined to 1.724 × 10^7^ (A), 5.261 × 10^7^ (B), 1.021 × 10^8^ (E) copies of *B. bassiana* DNA/*μ*L at the 9^th^ dpi and 8.292 × 10^7^ (C), 9.076 × 10^7^ (D) copies of *B. bassiana* DNA/*μ*L at the 7^th^ dpi. The concentration of *B. bassiana* DNA rebound at the 11^th^ dpi and reached 5.381 × 10^7^ (A), 1.156 × 10^8^ (B), 1.474 × 10^8^ (C), 1.553 × 10^8^ (D), and 1.615 × 10^8^ (E) copies of *B. bassiana* DNA/*μ*L at the 21^st^ dpi and then remained stabilized. Although the concentration of *B. bassiana* was different, the stability of *B. bassiana* DNA on Chinese cabbage leaves surface showed roughly the same trend, which showed a decreasing trend from the 1^st^ dpi to the 9^th^ dpi (except (A), (B), and (E)), all concentrations showed an increasing trend from the 11^th^ dpi to the 19^th^ dpi and then remained stabilized at 21^th^ dpi to the 29^th^ dpi. This experiment was repeated twice. *B. bassiana* DNA was not detected in the negative control samples.

### 4.6. Stability of Different Number Spores of *B. bassiana* DNA in *P. rapae* at 28°C

**Figure 5:**
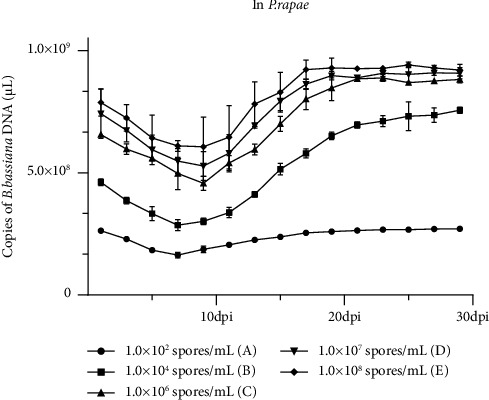
Stability of *B. bassiana* DNA in *P. rapae* at 28°C as determined by qRT-PCR. Three technical replicates were used for each sample, and the experiment was repeated twice. Statistical significances were determined by DMRT, *p* ≤ 0.05.

After the different number of spores of *B. bassiana* spraying on cabbage leaves at 12 hours, the treated leaves were fed to *P. rapae*. Then, the assayed *P. rapae* were analyzed for *B. bassiana* DNA concentrations by real-time PCR. Results shown in Figure 5 indicated that the stability of *B. bassiana* DNA were 1.633 × 10^8^ (A), 2.854 × 10^4^ (B) copies of *B. bassiana* DNA/*μ*L at the 7^th^ dpi; 4.574 × 10^8^ (C), 5.276 × 10^8^ (D), and 6.063 × 10^8^ (E) copies of *B. bassiana* DNA/*μ*L at the 9^th^ dpi. The concentration of *B. bassiana* DNA rebound at the 11^th^ dpi and, respectively, reached 2.638 × 10^8^ (A), 6.953 × 10^8^ (B), 8.839 × 10^8^ (C), 8.887 × 10^8^ (D), and 9.263 × 10^8^ (E) copies of *B. bassiana* DNA/*μ*L at the 21^st^ dpi. The mortality rate of *P. rapae* treated with different spores at 21^st^, respectively, reached 15% (A), 50% (B), 65% (C), 90% (D), and 95% (E), the concentration of 1.0 × 10^6^ (C) spores/mL reached 65% at the 19^th^ dpi; the concentration of 1.0 × 10^7^ (D) spores/mL reached 50% at the 13^th^ dpi and reached 90% at the 21^st^ dpi; the concentration of 1.0 × 10^8^ (E) spores/mL reached 50% at the 14^th^ dpi, and reached 90% at the 19^st^ dpi. This experiment was repeated twice. *B. bassiana* DNA was not detected in the negative control samples.

## 5. Conclusion and Discussion

In this study, we identified a pair of PCR primers specific for the *B. bassiana* 16s rDNA and established a sensitive and reliable PCR and a real-time PCR method for the detection of *B. bassiana* in various substrates. The sensitivity of the detection technology was 4.337 × 10^4^ copies of plasmid/*μ*L. Using this method, we can now reliably monitor *B. bassiana* population dynamics in *B. bassiana* preinoculated soil or cotton leaves samples. Our results indicated that during the 1^st^ to the 9^th^ dpi, *B. bassiana* DNA concentration declined in all substrates. According to our understanding, this decline was caused by the massive death of initially inoculated *B. bassiana*. The DNA concentration started to increase sharply after the 9^th^ till 11^th^ dpi. It is possible that this increase represents a rapid regrowth of *B. bassiana*, survived from the treatments. From the 11^th^ to the 29^th^ dpi, *B. bassiana* DNA concentrations remained high in all three substrates, suggesting that the *B. bassiana* population had reached the maximum level under the assayed conditions. In this study, *B. bassiana* DNA was not detected in any negative control samples and thus, the identified PCR primers produced the *B. bassiana* DNA specific bands when electrophoretically analyzed.

Different number spores of *B. bassiana* applied on Chinese cabbage result shown that the stability of *B. bassiana* DNA are consistent when plants treated with 1.0 × 10^6^, 1.0 × 10^7^, and 1.0 × 10^8^ spores/mL. But the mortality rate of *P. rapae* treated with different spores at 21^st^, respectively, reached 15%, 50%, 65%, 90%, and 95%, the concentration of 1.0 × 10^6^ spores/mL reached 65% at the 19^th^ dpi; the concentration of 1.0 × 10^7^ spores/mL reached 50% at the 13^th^ dpi and reached 90% at the 21^st^ dpi; the concentration of 1.0 × 10^8^ spores/mL reached 50% at the 14^th^ dpi, and reached 90% at the 19^st^ dpi. With the increase in the initial spores of *B. bassiana*, the mortality of *P. rapae* gradually increased. At low spores (1.0 × 10^2^ spores/mL and 1.0 × 10^4^ spores/mL), the death rate was approximately 15–50%. At high spores (1.0 × 10^6^, 1.0 × 10^7^, and 1.0 × 10^8^ spores/mL), the death rate was 65–95%. The results indicated the effect of killing insects was obviously increased with increasing the concentration of *B. bassiana*, whereas considering the cost-effectiveness, the 1.0 × 10^7^ spores/mL of *B. bassiana* was the most suitable.

Since the invention of real-time PCR, this technology has been widely applied to molecular biology studies [28]. Currently, PCR, and real-time PCR are the most popular technologies for plant pathogen diagnosis [29, 30] and host gene expression analysis. The potential of this technology for the assays on the stability of the inoculated biocontrol agents remained largely unknown. The main reason for this is mainly due to the lack of specific PCR primers. Current studies on *B. bassiana* field applications focused mainly on the development of antibiotic-resistant strains [31–33]. The development of antibiotic-resistant *B. bassiana* is, however, time-consuming and the resulting resistance strain(s) might become attenuated after a few generations. In contrast, understanding the environmental impacts on *B. bassiana* stability and recolonization behavior in the field can facilitate the design and establishment of more effective insect pest management strategies and thus achieving a better control effect. This PCR detection technology may also be modified for other biocontrol agents. In summary, this study has established a fast, low-cost, and reliable method for the determination of *B. bassiana* stability and recolonization in various substrates under the controlled conditions.

## Data Availability

The data that support the findings of this study are openly available at https://figshare.com/s/b65294b7548715e34c85.

## Abbreviations

*B. bassiana*: *Beauveria bassiana*
dpi: Day of post inoculation
*P. rapae*: *Pieris rapae*
PCR: Polymerase chain reaction.

## Supplementary Materials

Supplementary Materials Supplementary Figure S1: gradient PCR products separated in an agarose gel after electrophoresis. Supplementary Figure S2: standard curve of qRT-PCR. Supplementary Figure S3: qRT-PCR curves determined using different concentrations of plasmid DNA. Supplementary Table 1: six sets of PCR primers used in this study.
