# Supplementary material for: A Sensitive Method for Detecting Beauveria bassiana, an Insecticidal Biocontrol Agent, Population Dynamics, and Stability in Different Substrates
Source: Can J Infect Dis Med Microbiol. 2023 Aug 25;2023:9933783. doi: 10.1155/2023/9933783 (PMC10473894; doi:10.1155/2023/9933783)
Supplement: Supplementary Materials — Supplementary Figure S1: gradient PCR products separated in an agarose gel after electrophoresis. Supplementary Figure S2: standard curve of qRT-PCR. Supplementary Figure S3: qRT-PCR curves determined using different concentrations of plasmid DNA. Supplementary Table 1: six sets of PCR primers used in this study. [file 9933783.f1.zip › Supplemental Figure.docx]

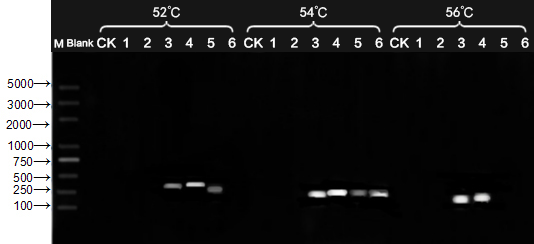


**Supplementary Fig. 1** Gradient PCR products separated in an agarose gel after electrophoresis. The annealing temperatures used in this study were 52, 54 and 56 ℃. Lanes 1, 2, 3, 4, 5 and 6 are PCR products amplified using different PCR primer sets. Each PCR reaction used 1 μL diluted plasmid DNA sample. Sizes of the DNA ladder are indicated on the left side, and the size of the expected PCR product is about 230 nucleotides. Lane CK indicates a PCR reaction without plasmid DNA and is used as a negative control.


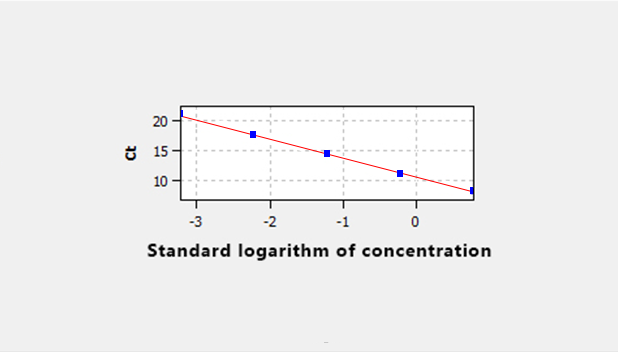


**Supplementary Fig. 2** Standard curve of qRT-PCR. Through PCR amplification assays, a standard curve of PCR was determined as Y = -3.26X+10.53, R^2^ = 0.99942, and the qRT-PCR amplification efficiency = 1.03


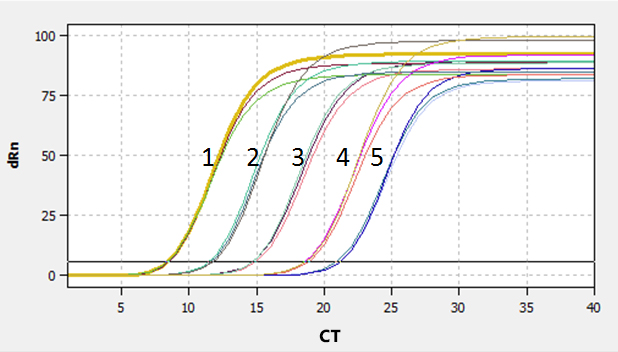


**Supplementary Fig. 3** QRT-PCR curves determined using different concentrations of plasmid DNA. Curve 1 to 5 were obtained using 4.337×10^8^, 4.337×10^7^, 4.337×10^6^, 4.337×10^5^, 4.337×10^4^ copies of plasmid DNA/μL.
