# Supplementary material for: A Sensitive Method for Detecting Beauveria bassiana, an Insecticidal Biocontrol Agent, Population Dynamics, and Stability in Different Substrates
Source: Can J Infect Dis Med Microbiol. 2023 Aug 25;2023:9933783. doi: 10.1155/2023/9933783 (PMC10473894; doi:10.1155/2023/9933783)
Supplement: Supplementary Materials — Supplementary Figure S1: gradient PCR products separated in an agarose gel after electrophoresis. Supplementary Figure S2: standard curve of qRT-PCR. Supplementary Figure S3: qRT-PCR curves determined using different concentrations of plasmid DNA. Supplementary Table 1: six sets of PCR primers used in this study. [file 9933783.f1.zip › Supplemental Table.docx]

**S-Table1:** Six sets of PCR primers used in this study.

| Primer set umber | Primer name | Primer sequence (5' to 3') |
| --- | --- | --- |
| 1 | 195F | 5'ACAACGGATCTCTTGGCTCT3' |
|  | 330R | 5'TTCGAGCGTCATTTCAACCC3' |
| 2 | 169F | 5'CCTTGCGGCGTATTCAGAAG3' |
|  | 214R | 5'AGAGCCAAGAGATCCGTTGT3' |
| 3 | 30F | 5'CATCTTCTGAATACGCCGCA3' |
|  | 232R | 5'GGGTTGAAATGACGCTCGAA3' |
| 4 | 98F | 5'GGCATCGATGAAGAACGCAG3' |
|  | 333R | 5'GTATTACTGCGCAGAGGTCG3' |
| 5 | 75F | 5'TCAACAACGGATCTCTTGGC3' |
|  | 242R | 5'GGGAGGTCGAGGGTTGAAAT3' |
| 6 | 30F | 5'CATCTTCTGAATACGCCGCA3' |
|  | 232R | 5'GGGTTGAAATGACGCTCGAA3' |
